# Hydrogen Generation Using a CuO/ZnO-ZrO_2_ Nanocatalyst for Autothermal Reforming of Methanol in a Microchannel Reactor

**DOI:** 10.3390/molecules16010348

**Published:** 2011-01-07

**Authors:** Kuen-Song Lin, Cheng-Yu Pan, Sujan Chowdhury, Mu-Ting Tu, Wan-Ting Hong, Chuin-Tih Yeh

**Affiliations:** Fuel Cell Center, Department of Chemical Engineering and Materials Science, Yuan Ze University, Chung-Li 320, Taiwan

**Keywords:** washcoat, microchannel, hydrogen generation, zirconia sol, autothermal reforming, fuel cell

## Abstract

In the present work, a microchannel reactor for autothermal reforming of methanol using a synthesized catalyst porous alumina support-CuO/ZnO mixed with ZrO_2_ sol washcoat has been developed and its fine structure and inner surface characterized. Experimentally, CuO/ZnO and alumina support with ZrO_2_ sol washcoat catalyst (catalyst slurries) nanoparticles is the catalytically active component of the microreactor. Catalyst slurries have been dried at 298 K for 5 h and then calcined at 623 K for 2 h to increase the surface area and specific pore structures of the washcoat catalyst. The surface area of BET N_2_ adsorption isotherms for the as-synthesized catalyst and catalyst/ZrO_2_ sol washcoat samples are 62 and 108 ± 2 m^2^g^−1^, respectively. The intensities of Cu content from XRD and XPS data indicate that Al_2_O_3_ with Cu species to form CuAl_2_O_4_. The EXAFS data reveals that the Cu species in washcoat samples have Cu-O bonding with a bond distance of 1.88 ± 0.02 Å and the coordination number is 3.46 ± 0.05, respectively. Moreover, a hydrogen production rate of 2.16 L h^−1^ is obtained and the corresponding methanol conversion is 98% at 543 K using the CuO/ZnO with ZrO_2_ sol washcoat catalyst.

## 1. Introduction

Recently, fuel cell systems have become promising portable power source candidates [1]. Applications for mobile energy based on the uses of methanol and hydrogen are spreading enormously with the exponential increase in electrical energy consumption demand. The lower energy density of conventional batteries makes them incapable of fulfilling these huge energy requirements [1,2]. The delivery of hydrogen to automotive sector and consumer electric devices is difficult and expensive, but these difficulties can be eliminated by using on-board reforming of liquid fuels in the respective applications [2]. Microchannel methanol reforming reactors for fuel cell applications is very suitable for the production of hydrogen compared to conventional systems like packed-bed reactors due to their size and weight factors [3,4,5,6,7]. In addition, microreactors have the advantages of improving heat and mass transfer, due to more precise control of reaction temperatures within the flammable region that leads to reduced hot spots and higher surface-to-volume ratios [8]. Moreover, high catalytic activity and selectivity of the catalyst coatings are crucial to the practical applications of microchannel methanol steam reforming reactors in integrated fuel cell systems. Therefore, procedure of catalytic slurry preparation for the catalyst washcoat needs to be optimized [9,10,11].

Methanol is one of the most promising sources of hydrogen for fuel cell applications as is displays the advantages of high energy density, easy availability, and safe handling/storage materials [12,13]. In the literature, CuO/ZnO–Al_2_O_3_ coated on a microchannel reactor is one of the most used catalysts for hydrogen generation by Steam Reforming of Methanol (SRM) [14,15,16]. Different reactions with different enthalpies occur in a microchannel reactor to produce hydrogen rich gas from methanol under standard conditions, as represented in Table 1 [6,7]. SRM is the main reforming reaction that provides the stoichometric conversion of methanol to hydrogen. The overall methanol decomposition reaction can be regards as the effect of the conversion of CO, CO_2_, H_2_O and the water gas shift (WGS) reaction. Whereas, the demand of hydrogen generation using SRM for a fuel cell can be met by a microstructured compact reformer. SRM is one of the attractive approaches currently on the rise because of higher energy density and relatively low reforming temperatures (450–600 K). In addition, methanol steam reforming produces a relatively small amount of carbon monoxide at low temperature; and carbon monoxide is known to be very poisonous for the Pt catalyst in a proton exchange membrane fuel cell (PEMFC) system [13]. Therefore, there has been considerable interest in the development of catalytic performance of microreactors equipped with microchannels.

**Table 1:** Some reactions inside a microreformer and their standard enthalpies of formation [8,9].

Reaction	Scheme	Δ *H* 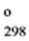 (kJ mol^−1^)
Methanol decomposition (MD)	CH_3_OH → 2 H_2 _+ CO	+92.0
Water-gas shift reaction (WGS)	CO + H_2_O → H_2 _+ CO_2_	−42.6
Steam reforming of methanol (SRM)	CH_3_OH + H_2_O → 3 H_2 _+ CO_2_	+49.4
Partial oxidation of methanol (POM)	CH_3_OH + ½ O_2_ → 2 H_2_ + CO_2_	−192.2
Total oxidation of methanol (TOM)	CH_3_OH + 3/2 O_2_ → CO_2_ + 2 H_2_O	+673.2

The effects of Cu/Zn ratio and the impact of metal loading have been investigated to optimize the catalysts for a SRM reaction. However, the catalyst incorporation onto the microchannel is difficult [17,18,19]. The preparation procedure is influenced by low interactions between metallic reactor surfaces and catalyst slurries. To investigate the SRM reaction several coating techniques have been applied for microchannels such as chemical vapor deposition (CVD), physical vapor deposition (PVD), anodic oxidation, sol-gel process, washcoating, sputtering, hydrothermal synthesis of zeolites, and electro-deposition [18,19,20]. Washcoat techniques in the microreformer with the use of optimistic gelation of boehmite slurries between the thicknesses from 1 to 25 μm are an attractive route for portable methanol reforming processes. According with Germani *et al*. [17] the binder play a major role on the basis of: (i) slurry viscosity being affected by its chemical structure and molecular weight, (ii) coating adhesion, and (iii) catalytic activity by re-dispersion of the active phase because formation of metal complexes influences the catalytic activities in the WGS reaction. In addition, the washcoat method has some advantages to obtain the utmost performance for the SRM reaction based on the resulting porosity and surface structures. These properties can also be changed by the concentration differences for pre-sols and the treatment procedures such as drying and calcination temperatures [21,22]. Stutz and Poulikakos [23] have indicated that a highly catalytically active thinner washcoat layer is not sufficient to process the inflowing reactants. Therefore, the main objectives of the present work were to prepare a proper CuO/ZnO-Al_2_O_3_ catalyst washcoat with ZrO_2_ sol and to investigate the autothermal reforming of methanol in a microchannel reactor for a fuel cell system. In addition, the fine structures of catalyst washcoats were also identified and characterized using scanning electron microscopy (SEM), optical microscopy (OM), X-ray diffraction (XRD), BET nitrogen adsorption isotherms, X-ray photoelectron spectroscopy (XPS), X-ray absorption near edge structure (XANES) or extended X-ray absorption fine structure (EXAFS) spectroscopy.

## 2. Results and Discussion

### 2.1. Morphology and crystalline structure identification

**Figure 1:**
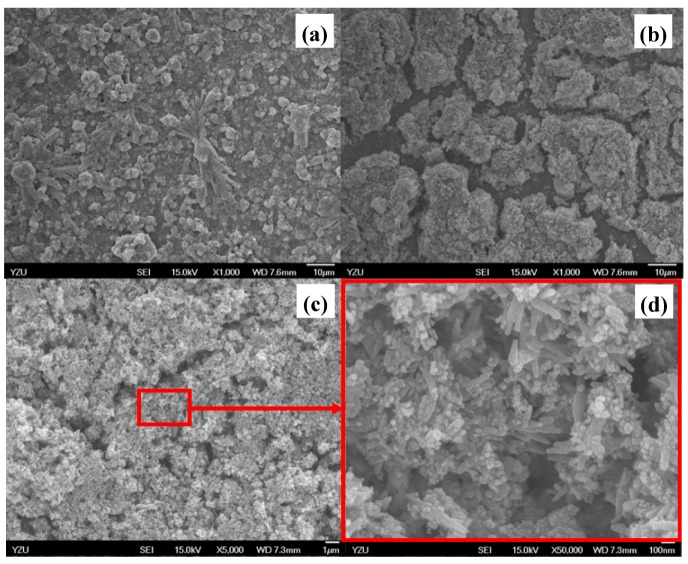
SEM microphotos of: **(a)** fresh as-synthesized CuO/ZnO-Al_2_O_3_ catalyst, and catalyst washcoated on Fe-Cr-Ni stainless steel 304 foil under the conditions of: **(b)** drying at 298 K for 5 h, **(c)** and **(d)** calcination at 623 K for 2 h, respectively.

Development of washcoats for microreactor applications has been conducted in recent years. Cu-based catalysts are mostly used for methanol reforming in microreactors considering their better reactivity and selectivity [24,25,26]. Coordinative vacancies of the Cu could be involved in the chemical reaction to provide the catalytic activity, while Zn(II) performs as a stabilizer for Cu surface area and Al(III) enhances the Cu-dispersion and catalytic stability [21,25,26]. According to the SEM images, significant morphological differences are observed between the as-prepared catalyst and the slurries as seen in Figure 1(a) to 1(d). In addition, the as-synthesized catalyst gradually changes in color from deep brown to blue after the addition of zirconia sol solution and isopropyl alcohol (IPA). This observation is consistent with the EDS measurement results. In fact, absorbance of water molecules shrinks the electron density of Cu(II) ions. Therefore, copper species are associated with *d-d* transition state and Cu(II) ions coordination state, as reflected by the color changing behavior for washcoat slurry materials. Thus, this confirms the rough washcoat surfaces are consistent with the appearance of a porous material [11]. Furthermore, the microstructure of the washcoat formed a needle-shaped surface at 623 K with calcination in the synthetic process, as shown in Figure 1(c) and 1(d), while the washcoat surface has been transformed into a brown color due to desorption of water molecules at the higher temperature of 623 K.

Generally, it is well known that the mechanical stability and adhesion to the SS 304 substrates are crucial due to the fact that detachment of ultrafine particles can easily lead to the blockage of the microchannels due to their small dimensions [20,21,22,23]. Furthermore, a porous support, such as ZrO_2_ layer was washcoated onto the microchannels to enhance the adhesion between the catalyst washcoat layer and SS 304 substrate. 

**Figure 2:**
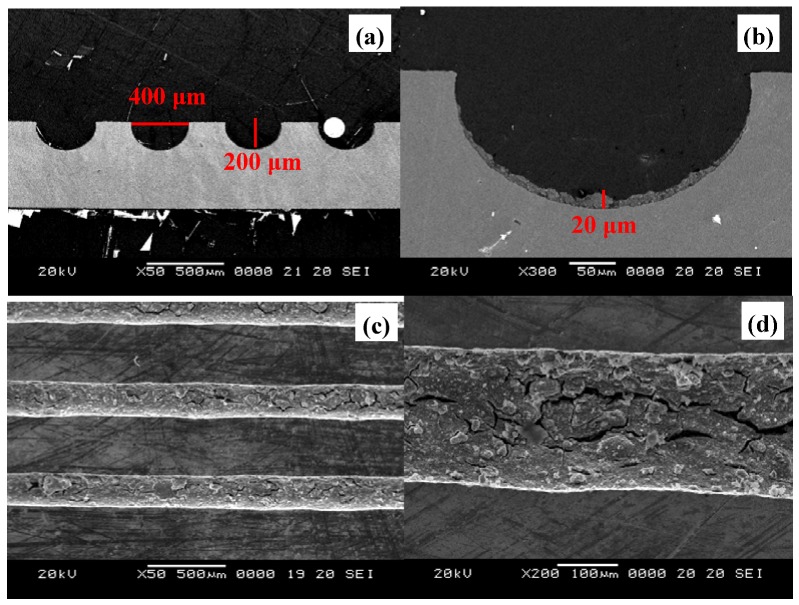
SEM microphotos of coating layers inside microchannels for **(a)** before washcoating (top view), **(b)** after washcoating (top view), **(c)** after washcoating (view of side cross-section) and **(d) **the fine porous structures of washcoat catalysts (portion of cross-section view).

Zirconia sols contain ceramic materials that have superior adhesive properties than other inorganic materials calcined in higher temperatures [21]. ZrO_2_ sol solutions were diluted with IPA solvent to adjust the viscosity to a suitable value. As shown in Figure 2(a), the microchannel dimensions were etched precisely to 400 μm (width) × 200 μm (depth). No cracks were found in the 20 μm in thickness catalyst washcoat layer, even after ultrasonic treatment for over 1 h, as shown in Figure 2(b). In addition, thermal treatment of the catalyst washcoat made of ZrO_2_ sol solution was performed in the range of 573–773 K. The structures seen in the cross-section image observations were strongly affected by the cutting treatment of the plate samples. The improper stress or bending of the plates may cause serious damages to the catalyst washcoat layers, as displayed in Figure 2(c), Figure 4(d). The catalyst washcoat layers on the microchannels were also adhered strongly and applicable for the subsequent SRM reactions.

### 2.2. XRD pattern analyses

**Figure 3:**
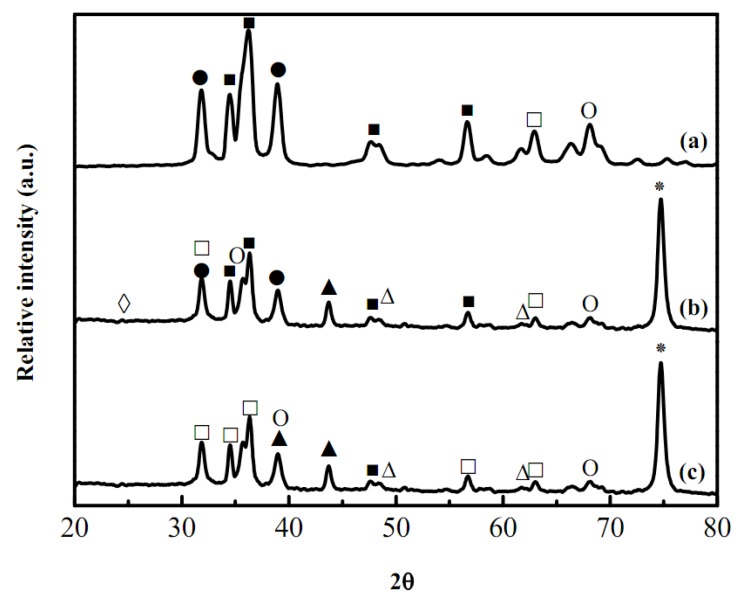
XRD patterns of washcoat catalysts coated mixed with ZrO_2_ sols and as-synthesized CuO/ZnO-Al_2_O_3_ catalyst for a microreactor on Fe-Cr-Ni stainless steel 304 foil of **(a) **fresh as-synthesized CuO/ZnO-Al_2_O_3_ catalyst, **(b)** after drying at 353 K, and **(c)** after calcinations at 623 K; (●: Cu; O: CuO; ■: Zn; □: ZnO; ▲: CuAl_2_O_4_; ∆: CuAlO_2_; ◊: ZrO_2_; *: SUS 304).

Recent studies of washcoat properties have faced the challenge of understanding interior and the surface structures for the proposed use. X-Ray diffraction patterns for the washcoat catalyst are shown in Figure 3. It shows that the fresh as-synthesized catalysts containing Zn, Cu or Al species could be identified by their peak positions and the standard intensities. In Figure 3(c), the weak peaks at Bragg angles 2*θ* of 32.5 and 35.5° was attributed to CuO and the characteristic peaks of ZnO were also seen at 2*θ* of 31.8, 34.5, and 36.3°, respectively, while the characteristic strong peaks of stainless steel at 2*θ* of 43.7 and 79.8° were seen in Figure 3(a) and 3(b), respectively [from the Joint Committee on Powder Diffraction Standards (JCPDS) database]. Catalyst powder was prepared using the same precursor as applied onto the microchannels for XRD analysis to discriminate and determine the attribution of these characterization peaks. Figure 3(b) shows the fresh as-synthesized samples after drying and revealed that the oxidation states of the Cu and Zn (pH of slurry was maintained at around 4 in the presence of nitric acid and CuO with ZnO were dissolved at pH < 5 and < 7, respectively). After drying and calcination as shown in Figure 3(a) and 3(b), XRD patterns of as-synthesized washcoat catalysts coated with ZrO_2_ sol onto the microreactor indicated that the Cu and Zn species were oxidized to CuO and ZnO, respectively. Closer inspection of the XRD patterns revealed that the washcoat catalyst was in the monoclinic phase and thermodynamically stable at the room temperature. The intensity of reflexions assigned to CuO increased with increasing temperatures. In addition, eventually the deactivation of washcoat catalysts coated with ZrO_2_ sol and as-synthesized catalyst was caused due to the phase changes of zirconia sol mixed with the as-synthesized catalyst (CuO 40%, ZnO 50%, and Al_2_O_3_ 10%) and HNO_3(aq)_ at lower pH values. Furthermore, ZnO could be attributed with Al_2_O_3_ at 623 K for 2 h of calcination. The SEM microphotograph [Figure 1(d)] with higher magnification revealed that some fine particles with spikes on the catalyst surfaces related with ZnO species, so according to the SEM/EDS analysis, the morphology of the alumina and zirconia may be explained by the occurrence of a Zr-O-Al phase with the migration of zirconium ions to the subsurface layers of the support. In addition, dispersion of the Zr-O-Al phase onto the support was consistent with the higher disorder and could be involved with the thermal stability and better performance for a long-term microreactor operation.

### 2.3. Temperature program reduction measurement

**Figure 4:**
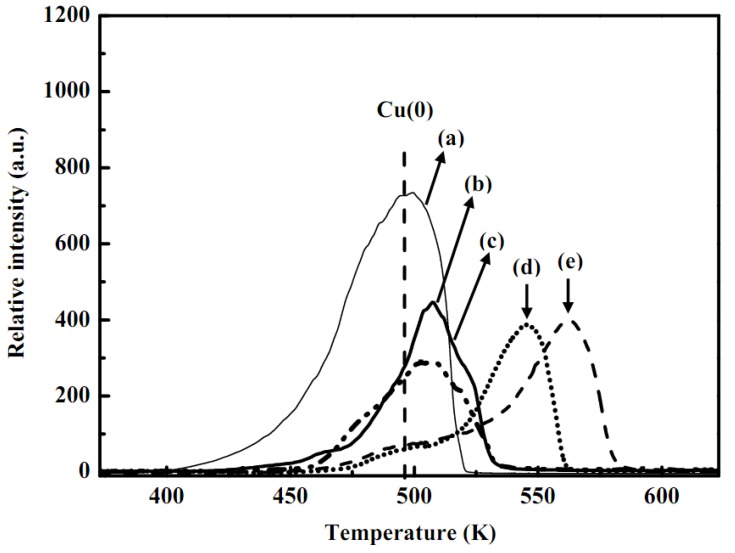
TPR curves of: **(a)** metallic copper, **(b)** fresh CuO/ZnO-Al_2_O_3_ catalyst, as synthesized CuO/ZnO-Al_2_O_3_ catalyst at pH 4 mixed with ZrO_2_ sols for stirring times of: **(c)** 30 min (S_C_20-R_C/B_10-pH4.0-St0.5), **(d)** 6 h (S_C_20-R_C/B_10-pH4.0-St6), and **(e)** CuO/Al_2_O_3_ standard (notes: Sc: solid content of (catalyst + binder) / total weight of (catalyst + binder + sol) (wt. %); R_C/B_: solid weight ratio of catalyst to binder (wt. %); pH: pH value of washcoat slurry; St: stirring time).

The TPR profile correspondence to the reduction of metallic copper and the copper oxide doped with alumina oxides or zinc oxides is displayed in Figure 4. The reducibility of the metallic Cu and CuO/Al_2_O_3_ were around 495 and 562 K, respectively as shown in Figure 4(a) and 4(e). An increase of the reduction temperature for CuO/Al_2_O_3_ sample was due to the effects of the linking bridge between Cu–Al interfaces. In addition, accumulation of the ZnO on the CuO/Al_2_O_3_ reduced the reductive temperatures and thus the linking bridge between Cu and Al was separated, as displayed in Figure 4(b). Catalyst samples showed no significant differences in the reduction temperatures with ZrO_2_ sols for stirring time (at pH = 4) from 30 min to 6 h, respectively, as shown in Figure 4(c) and 4(d). In addition, at longer stirring period of time would effect the dissolution of ZnO with consequent reduction of the CuO to Cu(0) or Cu(I), as represented in Figure 4(e). Therefore, particle size may increase after longer stirring of the ZrO_2_ sols with CuO/ZnO-Al_2_O_3_ catalyst at pH = 4. However, copper species are associated with d-d transition state as Cu(II) species due to the shorter period of stirring time cannot be destroyed the Zn(II) oxidation state as displayed by the similar trend seen in Figure 4(c).

### 2.4. BET surface area and pore volume distribution

**Figure 5:**
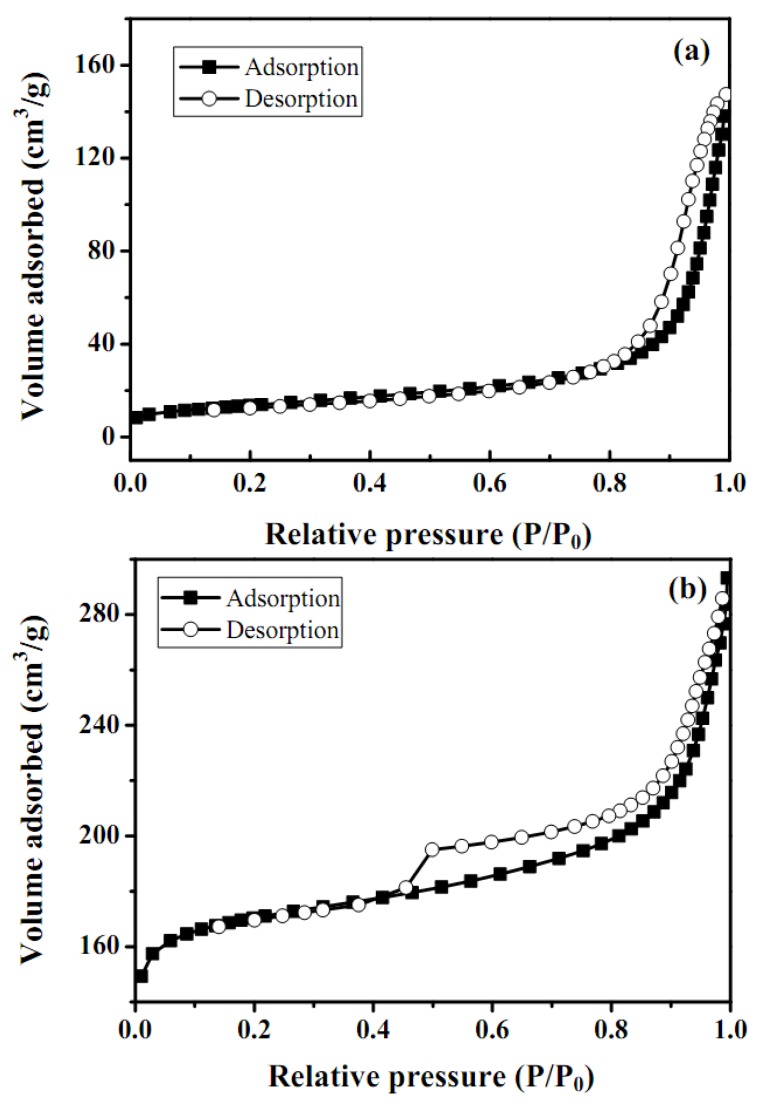
Nitrogen adsorption/desorption isotherms for: **(a)** as-synthesized catalysts and **(b)** as-synthesized catalytic mixtures with ZrO_2_ sols.

Nonspecific physical adsorption of the washcoat was carried out to measure the total surface area, pore size distribution for the as-synthesized catalyst and catalyst mixed with ZrO_2_ sol, as shown in Figure 4 and Figure 5, respectively. The surface area, pore size distribution of as-synthesized catalyst and catalyst/ZrO_2_ sol were calculated according to the adsorption data summarized in Table 2. A large surface area observed for as-prepared catalytic mixtures with ZrO_2_ sol and was considered a beneficial characteristic for a great variety of applications. Most of the microcrystalline structures of ZnAl_2_O_4_ were seen as fine particles, confirmed by the SEM analysis. The adsorption-desorption isotherms exhibited a hysteresis behavior and indicated that the specimens were mainly mesoporous. In general, a type IV hysteresis isotherm was obtained and represented in Figure 5. 

**Figure 6:**
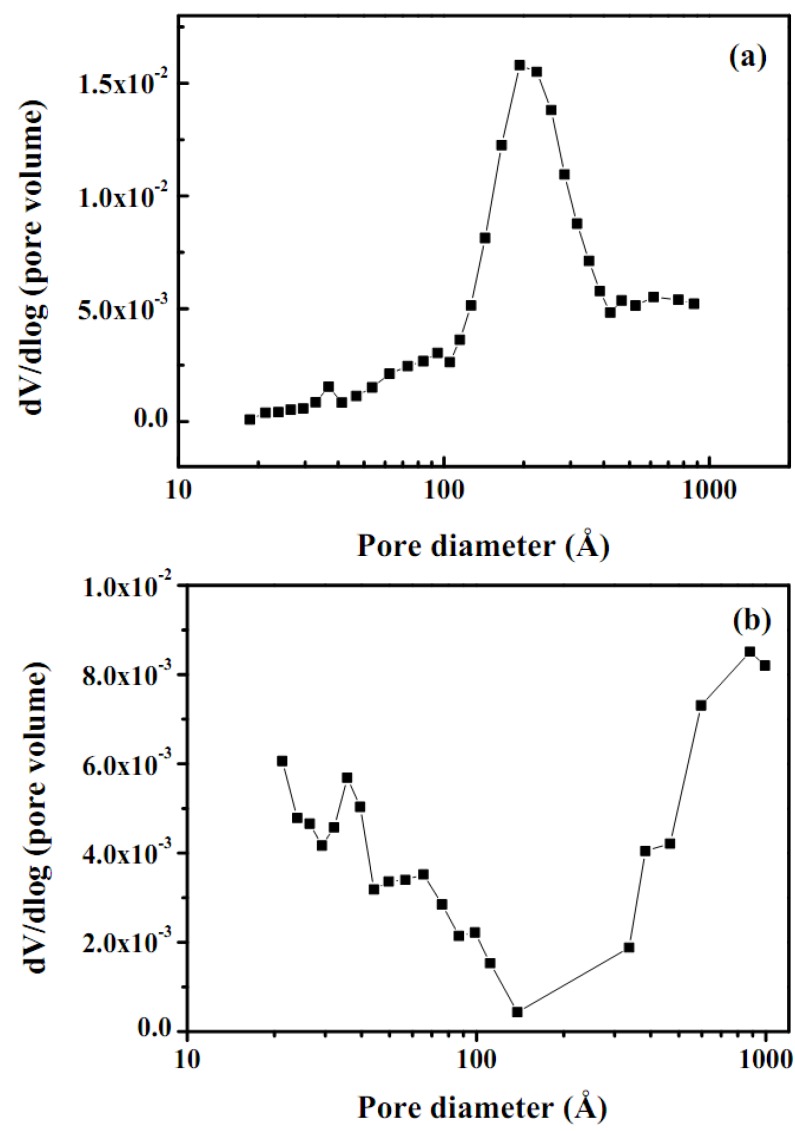
Pore size distribution for **(a)** as-synthesized catalyst and **(b)** as synthesized catalytic mixtures with ZrO_2_ sol.

Adsorption hysteresis was observed in the region of a relative pressure *P*/*P*_0_ above 0.4. A comparison between the shapes of the two isotherms in the pressure range of *P*/*P*_0_ 0–0.7 [Figure 5(a) and 5(b)] revealed a more pronounced hysteresis in the as-prepared catalytic mixture with ZrO_2_ sol. Furthermore, BET N_2_ adsorptions for as-synthesized catalyst and catalytic mixtures with ZrO_2_ sol washcoat were 62 and 108 ± 2 m^2^g^−1^, respectively. It also indicated that the addition of ZrO_2 _sol increased the higher surface area and porosity of the catalyst. The BJH method was employed to analyze the pore size distribution, which clearly showed that the porosities of the as-prepared catalyst were 30 and 70 % and that of the mixtures with ZrO_2_ sol were 10 and 90 %, respectively, as displayed in Figure 6 and Table 2.

**Table 2:** Specific surface area/pore size distribution of fresh catalysts and catalyst/ZrO_2_ sol mixtures using BET nitrogen isotherms and crystalline size of copper species.

Catalyst	S_BET_ *^a^*± 2	*V*_t_*^b ^*± 0.005	Pore size distribution		Crystalline size
	(m^2^ g^−1^)	(cm^3^ g^−1^)	*V*_micro_*^c^* (%)	*V*_meso_*^d^* (%)	Cu(111) (nm) ± 0.2
Fresh catalyst	62	0.080	0.025 (30)	0.059 (70)	8.7
Catalyst/ZrO_2_ sol mixture	108	0.160	0.017 (10)	0.145 (90)	8.1

*^a ^*S_BET_: specific surface area calculated using BET equation; *^b ^V*_t_: total pore volume estimated at a relative pressure of 0.98; *^c ^V*_micro_: micorpore volume determined using the Dubinin-Radushkevish equation; *^d ^V*_meso_: mesopore volume determined by the subtraction of micropore volume from total pore volume.

The mixture of microspores and mesopores contributed to the behavior of the washcoat samples and was involved in the hysteresis behavior. In general the washcoat behavior was consistent with the higher pore structure, and the condensation did not begin with an increasing pressure until it exceeds that corresponding to the effective radius of the interior. Moreover, after the interior pores were filled with nitrogen, then the pressure decreased to a value corresponding to the minimum radius of the neck before the pore was ready to be filled out. Since a washcoat provided a large surface area and porous structures, it had many active sites that would be useful for the catalytic SRM application.

### 2.5. XPS analyses and XANES/EXAFS measurement

The composition of the washcoat surface layer was investigated by means of the XPS technique. The changes in the chemical states for as-synthesized and used catalysts are shown in Table 3. The as-synthesized catalyst represented in Table 3 had a higher concentration of aluminum (38.1%) than zinc (33.7%) and copper (28.2%) on the surface. Three components observed (Table 3) in as-synthesized catalyst at 934.5 eV were assigned to CuO. Another Cu(II) species at 934.7 and 935.4 eV, respectively were related with either Cu(II) interaction with aluminum contained in the catalyst or hydroxyl groups at the ZnO surfaces [24,25,26,27]. CuO (86.6%) and Cu (42.5%) are the main species in the fresh catalyst and washcoat mixture, respectively, indicating that surface copper oxides were partially reduced to metallic Cu after the SRM reaction. In addition, a significant Cu(II) satellite peak was also observed at 943 eV. Aluminum oxidation was significantly lowered due to the fact the Zr was combined with the Al at higher temperatures and formed Zr-O-Al structures. This observation was similarly consistent with and confirmed by the SEM and XRD techniques.

**Table 3:** Composition and binding energy ((eV) O (*1S*) of Cu (*2P_3/2_*)) of copper species in fresh and CuO/ZnO/Al_2_O_3_ washcoat catalysts determined using XPS spectra.

Species	Cu		Cu_2_O		CuO
Cu (*2P_3/2_*)					
Bonding energy (eV)	932.7		932.5		933.7
*Concentration (%) (binding energy (eV))*					
Fresh CuO/ZnO/Al_2_O_3_	3.4 (932.7)		10.0 (932.6)		86.6 (934.5, 934.7, 935.4)
Cu/ZnO/Al_2_O_3 _+ ZrO_2_ mixture	42.5 (932.6)		32.6 (932.5)		24.9 (934.3, 934.6, 935,4)
**Species**	**γ–Al_2_O_3_**	**ZrO_2_**		**ZnO**	**CuO**
O (1S)					
*Concentration (%) (binding energy (eV))*					
Fresh CuO/ZnO/Al_2_O_3_	38.1 (531.0)	Not available		33.7 (530.4)	28.2 (529.9)
Cu/ZnO/Al_2_O_3 _+ ZrO_2_ mixture	10.1 (530.9)	2.2 (530.2)		30.0 (530.4)	47.7 (529.8)

Note: The catalytic slurry was synthesized by adding the ZrO_2_ sol solution to the prepared as-synthesized catalyst (CuO 40%, ZnO 50%, and Al_2_O_3_ 10%) and HNO_3(aq) _solution.

**Figure 7:**
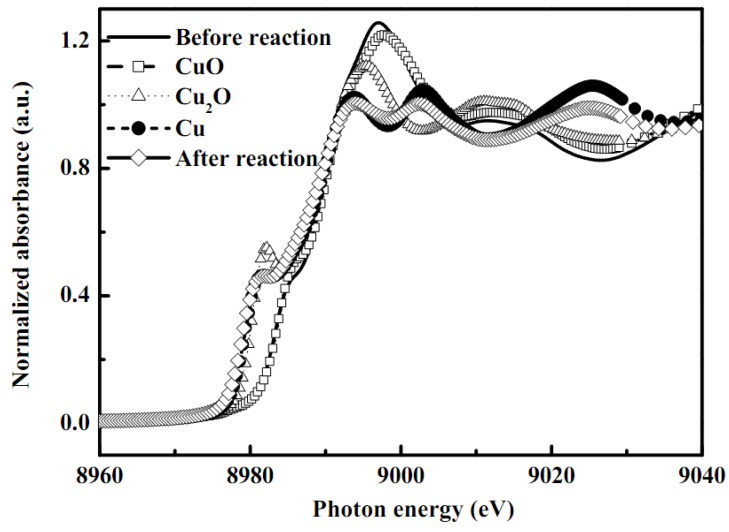
XANES spectra of copper standards and copper species in the as-synthesized CuO/ZnO-Al_2_O_3_ catalyst washcoat with ZrO_2_ sol before or after the steam reforming of methanol in a microreactor.

Generally, Cu K-edge EXAFS spectroscopy can provide information on the atomic arrangement of sorbents in terms of bond distance, coordination number, and kind of neighbors. In order to further investigate the structure of the copper species in the washcoat catalysts, X-ray absorption spectra of these catalytic samples were also performed (Figure 7 and Table 4). The XANES region of the X-ray absorption spectrum is very informative on both the oxidation and coordination states of Cu species. A high reliability of the EXAFS data fitting for Cu species in as-synthesized catalyst washcoat and before/after reforming reaction was obtained. The data were collected several times and standard deviation also calculated from the averaged spectra. Fourier transformation was performed on *k^3^*-weighted oscillations over the range of 2.2 and 10 Å^−1^. The XANES spectra of several different standards (Cu-foil, Cu_2_O, and CuO) and the different samples of porous alumina support CuO/ZnO with ZrO_2_ sol washcoat catalysts, and after reaction are shown in Figure 7.

The comparison is designed to determine the Cu in porous alumina support CuO/ZnO with ZrO_2_ sol washcoat catalysts to understand the catalytical effect(s) for the SRM reaction. The edge positions of standard Cu foil, Cu_2_O, and CuO are 8977.17, 8978.00, and 8980.76 eV, respectively and for as-synthesized catalyst and after SRM reaction the edge points were seen at 8980.76 and 8976.9 eV, respectively. It is found that the edge points of as-synthesized catalyst are very close to those of Cu(II) and its reduced form metallic Cu, *i.e*. Cu(0), after the reaction. However, the well-defined shoulders at 8985.12 eV are attributed to the 1s to 4p_xy_ transition that indicates the existence of Cu(II) species in the as-synthesized catalyst [24,25,26,27,28,29]. The edge of the first resonance in the XANES spectrum appears at 8997.16 eV due to the hydration of the Cu species. From the XANES spectra, the coexistence of Cu(II) in as-synthesized catalysts was identified and also trace Cu_2_O by-products. 

**Table 4:** Fine structural parameters of copper species in fresh and used Cu/ZnO/Al_2_O_3_ washcoat catalysts analyzed using EXAFS spectra.

Sample	Shell	CN*^a^*(±0.05)	R*^b^* (±0.02 Å)	Δσ^2^(Å^2^ )^c^
Fresh Cu/ZnO/Al_2_O_3_	Cu-O	3.46	1.88	0.0043
	Cu-Cu	3.95	2.69	0.0078
Used Cu/ZnO/Al_2_O_3_	Cu-O	2.86	1.93	0.0026
	Cu-Cu	4.09	2.13	0.0039
Elemental Copper (Cu)	Cu-Cu	11.05	2.54	0.0086
Curous oxide (Cu_2_O)	Cu-O	1.55	1.85	0.0029
Cupric oxide (CuO)	Cu-O	2.81	1.95	0.0043

Notes: ^a^ Coordination number; ^b^ Bond distance; ^c^ Debye-Waller factor

The Debye-Waller factors (Δσ^2^) in all EXAFS data were less than 0.01 Å^2^ which may indicate that the center Cu atoms are coordinated by Cu-O bonding. The standard Cu-O bond distances in CuO and Cu_2_O were 1.96 and 1.85 ± 0.02 , respectively, with coordination numbers of 2.18 and 1.55 ± 0.05, respectively. As-synthesized catalyst was consistent with the oxygen atom directly bonded to a centre Cu atom. It has the Cu-O bond length of 1.88 ± 0.02 with the coordination number 3.46 ± 0.05. After the SRM reaction, the structure of the as-synthesized catalyst was transformed into the metallic Cu(0) structure and the coordination number become to 4.09 ± 0.05 with the Cu-Cu bond distance 2.13 ± 0.02 Å. Moreover, it should be noted that surface copper oxides were partially reduced into metallic Cu after the SRM reaction. This leads to the conclusion that the attenuation of the EXAFS signal was due to the presence of amorphous material and was not related with the particle size.

### 2.6. Performance of the microreactor in a SRM reaction

**Figure 8:**
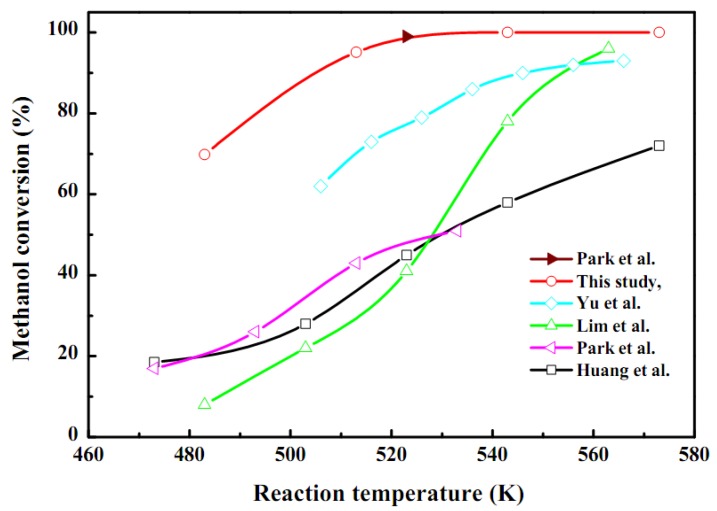
Comparision of methanol conversion of as-synthesized CuO/ZnO-Al_2_O_3_ catalyst washcoat with ZrO_2_ sol for autothermal reforming of methanol in a microreactor (steam/MeOH = 1.3; WHSV = 10 h^−1^; loading weight of washcoat catalyst = 10 mg/plate) and with literature works.

The SRM reaction was performed in order to investigate the as-synthesis catalyst activity in the stacked plate-type reactor as seen in Figure 8 and Table 5. A SRM performance test was carried out at 483–573 K with the same weight of coated catalyst packed in the microreactor. After the activation of the catalyst methanol started to decompose and the conversion was above 98% at 543 K. In addition, a higher temperature was required for higher methanol conversion, but decreased with an increase of feed flow rate and was related with the steam to carbon ratio [30,31,32,33,34,35,36,37,38,39]. According to Seo *et al*. [33], 90% methanol conversion was achieved at 533 K and the feed flow rate was 0.2 cm^3^ min^−1^. A hydrogen production rate of 2.16 L h^−1^ was obtained at a liquid methanol mixture feed rate of 0.05–0.2 mL min^−1^ with a WHSV of 10 h^−1^. Moreover, the selectivity of hydrogen and carbon dioxide was high enough and a typical dry gas composition of the present system was 73–75% H_2_, 24–26% CO_2_, and 0.1–1.2% CO gas products, respectively. This confirms that the as-synthesized catalyst coated onto the microchannel reactor participates in the SRM reaction and that is suitable for PEMFC applications for energy generation.

**Table 5:** Comparison of SRM performance over different CuO/ZnO/Al_2_O_3_/ZrO_2_ catalysts in the literature.

Catalyst	S/C^a^	WHSV^b^	S_CO_ (%)	T_C_^c^ (°C)	Binder	References
Cu_40_Zn_50_Al_10_	1.3	16.2	0.1–1.2	543 (98)	ZrO_2_	This work
Cu_50_Zn_33_Al_8_	1.1	54.0	2.90	533 (99)	Al_2_O_3_	Park *et al*. [15]
Cu_50_Zn_33_Al_8_	1.5	14.8	1.10	543 (80)	ZrO_2_	Lim *et al*. [21]
Cu_65_Zn_28_Ce_7_	1.3	8.3	2.05	565 (97)	Al_2_O_3_	Yu *et al*. [38]
Cu_48_Zn_48_Ce_4_	1.3	8.3	1.30	565 (98)	Al_2_O_3_	Yu *et al*. [38]
Cu_38_Zn_58_Ce_4_	1.3	8.3	1.60	565 (91)	Al_2_O_3_	Yu *et al*. [38]
Cu_48_Zn_48_Ce_4_	1.3	8.3	2.10	565 (88)	Al_2_O_3_	Yu *et al*. [38]
Cu_30_Zn_60_Al_10_	1.1	14.1	0.47	523 (74)	N.A.	Huang *et al*. [39]
Cu_40_Zn_50_Al_10_	1.1	14.1	0.49	523 (85)	N.A.	Huang *et al*. [39]
Cu_50_Zn_40_Al_10_	1.1	14.1	0.50	573 (89)	N.A.	Huang *et al*. [39]
Cu_60_Zn_30_Al_10_	1.1	14.1	0.49	573 (75)	N.A.	Huang *et al*. [39]

^a^S/C: steam to carbon ratio; ^b^WHSV: weight-hourly space velocity in mass methanol per time and mass of catalyst (g_MeOH_ h^−1^ g_cat._^−1^); ^c^(Temperature) (%) denotes “temperatures required for conversion of methanol at different percentage”; N.A. denotes “not available”.

## 3. Experimental

### 3.1. Microchannel reactor setup

**Figure 9:**
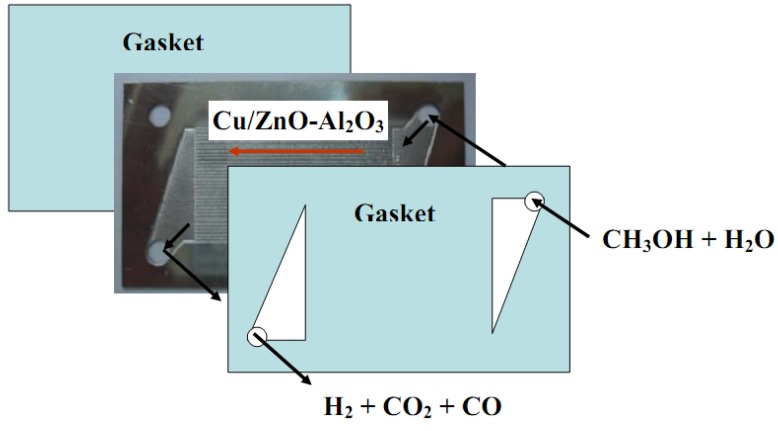
Schematic diagram for a typical steam reforming of methanol in a microreactor.

As shown in Figure 9, the microchannels in this microreactor were made of SS 304 stainless steel by a wet etching process and included a flow route for reactants and products. Usually two kinds of etching process are used, namely wet etching and dry etching. The wet etching process is performed by a wet chemical solution and dry etching process is carried out using a plasma. The wet etching process is relatively low cost, and has high reliability and output with excellent selectivity [18,19,20]. The size of the microchannel was 35 (L (Length)) × 0.4 (W (Width)) × 0.2 (D (Depth)) mm. The plate was designed with thirty-four microchannels with the plate of 60 (L) × 35 (W) × 0.6 (T) mm. According to Figure 9, CH_3_OH and O_2_ or water mixtures were passed through the right or left upper inlet and were distributed over the plates to the parallel microchannels sequentially. Finally, H_2_/CO_2_ mixtures were collected through the outlet at the bottom side. Several microchannel plates containing catalyst were stacked together and the catalytic performance was tested for a SRM reaction. A graphite gasket was used between microchannel plates for gas tightness and the whole reactor was contained in a stainless-steel housing.

### 3.2. Test of microreactor performance for methanol conversion

The tests of methanol conversion performance were conducted in a lab-scale five-plate microchannel reactor using a porous alumina support CuO/ZnO with ZrO_2_ sol washcoat catalysts. After the catalyst was applied in each plate then five plates were stacked together, well sealed and integrated in a housing. The total weight of as-synthesized catalyst coated on the five plates was 100 mg. A pre-mixture of methanol and water solution (1:1.3 molar ratio) was introduced to the microreactor using an automatic syringe pump with He carrier gas and then vaporized through a feed line heated at 455 K. The feed rate of methanol liquid reactant mixtures was 0.05–0.2 mL min^−1^ with 10 h^−1^ WHSV (weight hourly space velocity). For a steam-reforming process, pre-reduction was not necessarily required for every cycle. The reforming catalyst was activated *in-situ* by treatment with the vaporized feed mixtures at the reaction temperatures, whereby vaporized reactants were passed through the steam microreformer and were converted to a hydrogen-rich stream with trace CO. The SRM reaction was performed in the temperatures range from 483–573 K. The microreactor was enclosed and heated by an electrically heated jacket and operated under isothermal and isobaric conditions using a temperature controller. Temperatures were measured by a thermocouple placed inside the housing. The noncondensable streams of H_2_, CO, and CO_2_ gases generated in the microreactor were directly passed through a water cooler at 273 K and were measured with a totalizer. The liquid condensates were separated and collected in two knockout drums (50 and 100 mL) in series. The noncondensable gases were sampled and analyzed using an on-line FTIR and a GC/TCD [21].

### 3.3. Catalyst washcoat

In order to increase adhesion between the washcoating layer and the substrate, porous supports such as Al_2_O_3_ was coated onto the microchannels [40]. In this study, ZrO_2_ (Alfa Aesar, 20 wt. %) sol was used as a binder to enhance the adhesion between catalyst slurry and the SS 304 or substrates. Lim *et al*. [21] have reported that the dilute ZrO_2_ sol solution was better than a high concentration one to obtain uniform oxide layers. The catalytic slurry was synthesized by adding the ZrO_2_ sol solution to the prepared as-synthesized catalyst (CuO 40%, ZnO 50%, and Al_2_O_3_ 10%) and HNO_3(aq) _solution. As-synthesized homogeneous sol was prepared by adding reosmosis water (ROM) (Catalyst:ROM = 1:2) with continuous magnetic stirring at 500 rpm. An adequate viscous material was thus obtained. 

**Figure 10:**
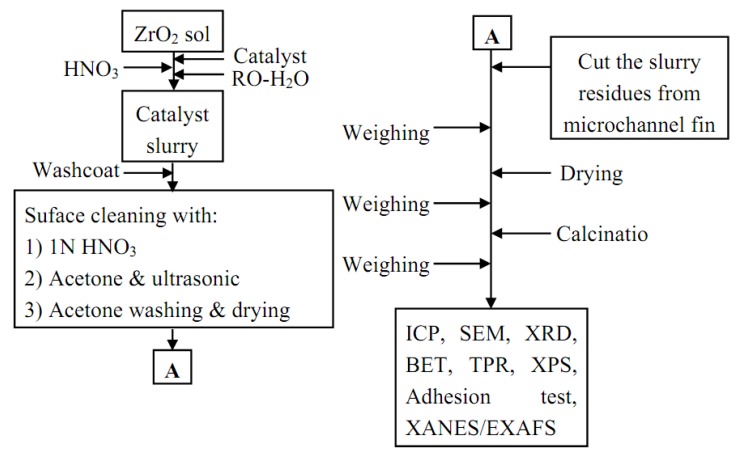
Procedure of catalyst slurry preparation and washcoating for the as-synthesized CuO/ZnO-Al_2_O_3_ catalyst washcoat with ZrO_2_ sol for steam reforming of methanol in a microreactor.

Diluted homogeneous solution was prepared by dropwise addition of HNO_3_ (J.T. Baker, 70%) and NaOH (Alfa Aeser, ACS grade) to maintain the pH value at 4. The homogeneous solution was continuously stirred for 30 min to 6 h to obtain the catalyst slurry. After, stainless steel substrate surfaces were cleaned with acetone/HNO_3(aq) _and then the catalyst slurry was washcoated on the surface of microchannels followed by drying at 298 K for 5 h. The process was repeated to give the desired weight of catalyst and the assembly was then calcined at 623 K for 2 h. After calcination, ZrO_2_ film was formed between catalyst and substrate. In addition, this ZrO_2_ film greatly improved the adherence of washcoating layer onto the microchannel. Catalyst layers were adhered strongly onto the microchannels for the catalytic applications. A washcoating procedure of catalyst slurry on substrates for a microchannel reactor is shown in Figure 10.

### 3.4. Catalyst characterization

The average concentrations of metals in washcoat catalysts were measured by inductively coupled plasma/mass spectroscopy (ICP/MS, PE-SCIEX ELAN 6100 DRC). The concentrations of Zr, Cu, Al or Zn species were calculated after calibrating the individual measured curves with their corresponding standard metal solutions at ten different concentrations bracketing the expected species. The oxidation state and molecular structure of as-synthesized catalysts surface were determined by X-ray photoelectron spectroscopy (XPS) using a VG Scientific Fison ESCA 210 spectrometer at the excitation energy of Mg K_α_ 1253.6 eV with 240 W [41]. Each XPS spectrum was taken in the preparation chamber of the spectrometer at 293 K for 60 min with a base pressure on the order of ~ 10^−9^ torr. The surface composition was determined after the subtraction of a Shirley-type background. The specific surface areas of as-synthesized CuO/ZnO–Al_2_O_3_ catalyst and the ZrO_2_ sols were measured according to the Brunauer-Emmer-Teller (BET) equation using the nitrogen adsorption isotherms at 77 K obtained with an ASAP2020 Micromeritics Surface Area and Porosity Analyzer. For the BET surface area measurements catalyst was scraped from the tube substrate and powdered to avoid any influence from the steel tubing. Prior to measurement, all samples were degassed at 423 K for 1 h. For the calculation of the BET surface areas, relative pressure range P/P_0_ of 0.05 to 0.2 was used. The pore radius distribution was determined by the method of Barrett, Joyner, and Halenda (BJH) method.

A conventional temperature programmed reduction (TPR) apparatus was used for the investigation of the reducibility of the Cu catalysts. TPR measurements were conducted using a flow rate of 30 mL min^-1^ of H_2_/Ar mixture (30 vol.% H_2_) at 1 atm. A catalyst sample of 0.5 g was tested each time and a heating rate of 10 K min^−1^ from 298 to 800 K was used. The morphologies, microstructures, and the elemental composition of the washcoat catalysts were determined on SEM/EDS (JEOL 5600) and OM (Olympus SZ61) instruments. Identification of the solid phases and crystalline structure of as-prepared catalyst samples was performed with by XRD analysis (Shimadzu Labx XRD–6000). All samples were therefore scanned from 20 to 70° (2θ) with a scan rate of 4° (2θ) min^−1^ and monochromatic CuK_α_ radiation. The specific peak intensities and recoded 2θ values were further identified by a computer database system (JCPDS). The adherence of the washcoating layer was evaluated by the method described in the literature [42,43,44]. In this study, the adhesion test was performed in an ultrasonic bath of D.I.-water for 30 min, and then between ultrasonic vibrations the mass balance was Calculated.

The XANES/EXAFS spectra were collected at the Wiggler beam line 17C1 at the NSRRC of Taiwan. The electron storage ring was operated with an energy of 1.5 GeV and a current of 100–200 mA. The double crystal monochromators (DCM) employing at either beamline selected X-rays with energy resolving power (E/ΔE) better than 7,000, sufficient for most XAS measurements. Data were collected in fluorescence or transmission mode with a Lytle detector [45] in the regions of the Zr (17,998 eV) K edges at room temperature. The EXAFS function was derived from the raw absorption data through pre-edge and post-edge background subtraction and then normalized with respect to the edge jump by using the AUTOBK program package after being *k*^3^-weigted (*k*-range = 2.5–12.5 ^−1^), where *k* is the photoelectron wave number. Local structural parameters such as the bond distance R, coordination number N, and Debye-Waller factor σ, for different coordination shells surrounding the absorbing atoms were obtained through non-linear least-square fitting routine. All the computer programs were implemented in the software package UWXAFS 3.0. The phase shifts and backscattering amplitudes were theoretically calculated using FEFF 8.20 code based on the crystallographic data of individual species [46,47]. Simulations were carried out to demonstrate the effects of the crystalline structure of the washcoat catalyst. Multiple scatterings were included in the calculations due to obtain the origin of the spectral characteristics of the zirconia structure included with the simplification of the Debye-Waller factors [45,46,47].

## 4. Conclusions

The objectives of the present study were to develop a SRM reactor to understand the fine structure and morphology of the as-synthesized catalysts. An as-synthesized CuO/ZnO with ZrO_2_ sol washcoat catalyst in a microchannel system was investigated using SEM or OM microphotos. The results revealed that the catalyst slurries dried at 298 K for 5 h and calcined at 623 K for 2 h showed significant increases in pore structure. Experimentally, the values of BET N_2_ adsorption for as-synthesized catalyst and catalyst/ZrO_2_ sol washcoat were 62 and 108 ± 2 m^2^g^−1^, respectively. It also indicates that organic templates in the slurry are burned and removed from the porous structures during the calcining processes. According with the XRD patterns, Cu and Zn species were oxidized to CuO and ZnO, respectively, at higher temperature and a thermally stable monoclinic phase was observed at room temperature. In addition, the XANES or EXAFS spectra identified the oxidative state and fine structures of the Cu species. The EXAFS data revealed that a Cu central atom has a Cu-O bonding with a bond distance of 1.88 ± 0.02 and its coordination number is 3.46 ± 0.05. A hydrogen production rate of 2.16 L h^−1^ was obtained at a feed rate of methanol liquid mixtures of 0.05–0.2 mL min^−1^ with a WHSV of 10 h^−1^. Moreover, the methanol conversion was about 98% at 543 K. Therefore, the microchannel methanol reforming reactor has potential for the production of high purity hydrogen and final processing to make it suitable for an integrated PEMFC system.
